# Top-down modulation of unconscious 'automatic' processes: A gating framework

**DOI:** 10.2478/v10053-008-0032-2

**Published:** 2008-07-15

**Authors:** Markus Kiefer

**Affiliations:** University of Ulm, Department of Psychiatry, Germany

**Keywords:** automatic processes, unconscious perception, masked semantic, priming, masked response priming, top-down control

## Abstract

In classical theories of automaticity, automatic processes are usually thought to occur autonomously and independently of higher level top-down factors (e.g., Posner & Snyder, 1975). However, already Neumann (1984) pointed out that the cognitive system has to be configured in a certain way for automatic processes to occur. In extension of his work, I propose a gating framework to account for the influence of top-down factors such as attention, intention and task set on automatic processes such as masked response or semantic priming. It is assumed that task representations held in prefrontal cortex regulate the gain of neurons in visual and sematic association cortex thereby modulating the effects of unconsciously perceived masked stimuli on further ‘automatic’ information processing steps. In support of the postulated gating framework, recent studies demonstrated a top-down modulation of automatic processes. Behavioral and electrophysiological studies with the masked response priming and semantic priming paradigms show that masked priming effects crucially depend (i) on temporal attention to the masked prime, (ii) on intentions or action plans and (iii) on the task set active immediately before masked prime presentation. For instance, masked semantic priming was only observed when the preceding task set required the orientation to semantic word features, but not when it required orientation to perceptual word features. These results support the view that unconscious automatic processes are modulated by top-down factors. They are suggestive of a gating mechanism which orchestrates the conscious and unconscious information processing streams.

## Masked semantic priming as an index of automatic processing

The effect of unconsciously perceived masked stimuli on the processing of subsequently presented visible stimuli is considered to be a prototypical example of an automatic process because the influence of strategic processing mechanisms can be ruled out. While the direct engagement of strategic processing is very unlikely during conditions of unconscious perception, I will show later in this article that this does not exclude the possibility of indirect modulatory influences of top-down mechanisms on automatic processing. In this article, I will focus upon automatic processes elicited by unconsciously perceived stimuli because in conditions of unconscious perception it can be ensured that processing occurs ‘automatically’ without any contribution of intended, strategic processes. This does not preclude the possibility that consciously perceived stimuli can also trigger automatic processes (e.g., Hommel, 2000). However, for consciously perceived stimuli it is difficult to rule out that controlled processes also contribute (see also the classification of semantic priming mechanisms below).

In this section, I will give a brief overview of the (masked) semantic priming paradigm and its application to investigate automatic semantic processes before I move on to discuss top-down influences on automatic processing. During the last decades, convincing evidence has been accumulated that the semantic meaning of masked words that cannot be consciously identified is activated and can influence processing of subsequently presented stimuli (semantic priming; for an overview, see Kiefer, 2002a). While it is well accepted that unconsciously perceived masked stimuli can prime an associated motor response (response priming; see Klotz & Neumann, 1999; Vorberg, Mattler, Heinecke, Schmidt, & Schwarzbach, 2003), it has been questioned that unconsciously perceived masked stimuli are processed also at the level of semantic meaning (Abrams & Greenwald, 2000; Damian, 2001). However, a variety of studies using the semantic priming paradigm, which is not compromised by confounding response priming effects, have reliably shown that semantic meaning is extracted from unconsciously perceived stimuli (e.g., Carr & Dagenbach, 1990; Kiefer, 2002b; Kiefer & Spitzer, 2000; for semantic priming during the attentional blink, see Rolke, Heil, Streb, & Henninghausen, 2001).

Complementary to response priming, the masked semantic priming paradigm is a powerful tool to study the nature of unconscious perception and - as we will see later – to study the modulatory effects on automatic processes for the following reasons: (i) Semantic priming rests on highly overlearned associations between concepts, which have been acquired within a long period of time (Anderson & Bower, 1973). Response priming, in contrast, depends on the congruency of stimulus-response (S-R) mappings established within the experiment or on the congruency of actions afforded by the stimulus (see Ansorge, Neumann, Becker, Kälberer, & Cruse, this volume; Kiesel, Kunde, & Hoffmann, this volume). Hence, automatic semantic priming presumably involves relatively hard-wired processing pathways between related concepts. Response priming, in contrast, is based on response competition evoked by the (in)congruency of S-R mappings between prime and target (Klinger, Burton, & Pitts, 2000). (ii) Semantic priming differs from response priming with regard to the underlying neural substrate. Semantic priming crucially depends on areas within the inferior and anterior ventro-medial temporal lobe, which belong to the ventral visual pathway (Nobre & McCarthy, 1995). The ventral pathway has an important role in object identification and conscious vision in general (Milner & Goodale, 1995). Response priming, in contrast, involves occipito-parietal regions, which belong to the dorsal pathway (Ansorge et al., this volume, Jaśkowski, Skalska, & Verleger, 2003). The dorsal pathway has been considered to be the neural substrate of unconscious visuo-motor processes subserving motor responses such as grasping movements (Milner & Goodale, 1995). Given these differences in functional neuroanatomy between semantic priming and response priming, it is of great interest to assess whether unconscious automatic processes underlying both forms of priming are governed by the same set of computational principles (see also the discussion in the final section of this article).

Semantic priming generally refers to the facilitation of a response to a target stimulus (e.g., a word) by a meaningfully related prime stimulus (Neely, 1991). In the masked semantic priming procedure, conscious perception of the prime is eliminated by displaying a pattern mask (e.g., a random sequence of letters) before and after the prime (for processes underlying masking, see for instance Scharlau, 2007, in this issue). Unconscious semantic activation is demonstrated when the masked prime word facilitates the processing of the target stimulus. Semantic priming has been frequently observed in lexical decision tasks in which subjects have to decide whether a target word (e.g., “lemon”) is a real word or a pseudoword. Reactions are faster and more accurate if a semantically related prime word (e.g., “sour”) precedes the target in comparison to a condition in which an unrelated word (e.g., “house”) precedes the target.

Two general cognitive mechanisms have been proposed to underlie semantic priming effects: Firstly, unconscious automatic spreading of activation and secondly, conscious strategic semantic processing (Posner & Snyder, 1975). According to the first cognitive mechanism, semantic priming reflects the automatic spread of activation in semantic networks. The presentation of a prime stimulus is thought to activate the corresponding conceptual representation in a semantic network, and activation spreads to semantically related nodes, hereby increasing their activation level. Hence, if a word denoting a related concept is presented, its recognition is facilitated. According to Posner and Snyder (1975) automatic spread of activation does not depend on capacity-limited attentional processes. In contrast, according to the second class of cognitive mechanisms (strategic semantic processing), semantic priming is the result of controlled attentional processes such as semantic matching or semantic expectation (for an overview, see Neely, 1991). By definition, strategic semantic processing depends on capacity-limited attentional resources (Posner & Snyder, 1975).

With visible prime stimuli, both automatic spreading activation and controlled priming processes usually contribute. For strategic semantic processing to occur, subjects must be aware of the presentation of the prime stimulus, semantic priming elicited by unconsciously perceived masked words exclusively arises from automatic spreading activation. Behavioural masked semantic priming effects have been reliably demonstrated in several studies (e.g., Kiefer, 2002b; Kiefer & Brendel, 2006; Marcel, 1983).

In addition to behavioural methods, semantic processes can also be studied with event-related brain potentials (ERPs), which have the advantage to capture cognitive processes online with a temporal resolution in the range of milliseconds and have been frequently shown to be more sensitive than behavioural measures (for a discussion, see Kiefer & Brendel, 2006). In ERP research on semantic processing, semantic priming effects are reflected by an amplitude modulation of the N400 ERP component. The N400 is a negative ERP deflection over the centro-parietal scalp, which specifically reflects semantic processing (Kutas & Hillyard, 1980). Studies using intracranial electrodes have suggested a generator in the anterior fusiform gyrus (Nobre & McCarthy, 1995). The significance of this brain area for semantic memory processes has also been shown in neuroimaging studies (e.g., Vandenberghe, Price, Wise, Josephs, & Frackowiak, 1996).

The N400 has been shown to be sensitive to semantic deviations with larger amplitudes for semantically incongruent words compared to congruent words at both the sentence (e.g., Friederici, Hahne, & Mecklinger, 1996; Kutas & Hillyard, 1984) and the word level (e.g., Bentin, McCarthy, & Wood, 1985; Kiefer, 2001, 2005). Using semantic priming paradigms, N400 amplitude to targets is attenuated for semantically related word pairs compared to unrelated word pairs, the so called N400 priming effect (e.g., Bentin, McCarthy, & Wood, 1985; Holcomb & Neville, 1990; Kiefer, Weisbrod, Kern, Maier, & Spitzer, 1998). There is evidence that the N400 potential is reliably modulated by masked words, which were not consciously perceived (Deacon, Hewitt, Chien-Ming, & Nagata, 2000; Kiefer, 2002b; Kiefer & Spitzer, 2000) and by words which were not available for report because they are presented during the attentional blink (Luck, Vogel, & Shapiro, 1996; Rolke, Heil, Streb, & Henninghausen, 2001; Vogel, Luck, & Shapiro, 1998). The results of these recent studies suggest that the N400 modulation also reflects automatic spread of activation.

These findings are in contrast to results from some earlier studies, which suggested that N400 amplitude is exclusively modulated by strategic semantic processing. In fact, there is some evidence that conscious or attentive processing of the prime is a prerequisite for N400 priming effects (for a review, see Deacon & Shelley-Tremblay, 2000): In an earlier masked priming study by Brown and Hagoort (1993), N400 priming effects were only obtained for visible, but not for masked primes, although behavioural priming effects were obtained in both conditions. N400 priming effects were found in a dichotic listening task for attended, but not for ignored prime words (Bentin, Kutas, & Hillyard, 1995). Finally, N400 priming effects were obtained only when an orienting task required semantic processing of the prime, but not when the task asked for visual processing of word features (Chwilla, Brown, & Hagoort, 1995). Hence, these studies suggest that attentive orientation to the prime is a prerequisite for N400 priming effects to occur.

**Figure 1.**
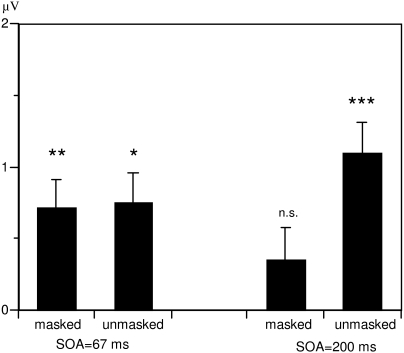
ERP priming effects. Absolute mean voltage difference between semantically unrelated and related word pairs (ERP priming effects) in the N400 time window at centro-parietal electrodes as a function of masking and prime-target SOA. Potentials were collapsed across hemispheres. This figure shows the qualitatively different time courses for unmasked and masked N400 priming effects (after Kiefer and Spitzer, 2000).

It has been proposed that masked N400 priming effects strongly depend on the interval between the onset of the prime word and the target (stimulus onset asynchrony, SOA) and that the use of the long SOA of 500 ms in the Brown and Hagoort (1993) study is one possible explanation for their failure to detect masked N400 priming effects (Deacon, Hewitt, Chien-Ming, & Nagata, 2000; Kiefer, 2002b; Kiefer & Spitzer, 2000). In fact, when varying the SOA systematically, Kiefer and Spitzer (2000) found masked N400 priming effects at an SOA of 67 ms, but not at an SOA of 200 ms. Unmasked N400 priming effects, in contrast, increased at the longer SOA (see Figure 1). This study shows that masked priming on the N400 ERP component can be obtained provided that prime and target stimuli appear in close succession, but decays rapidly within about 200 ms.

**Figure 2.**
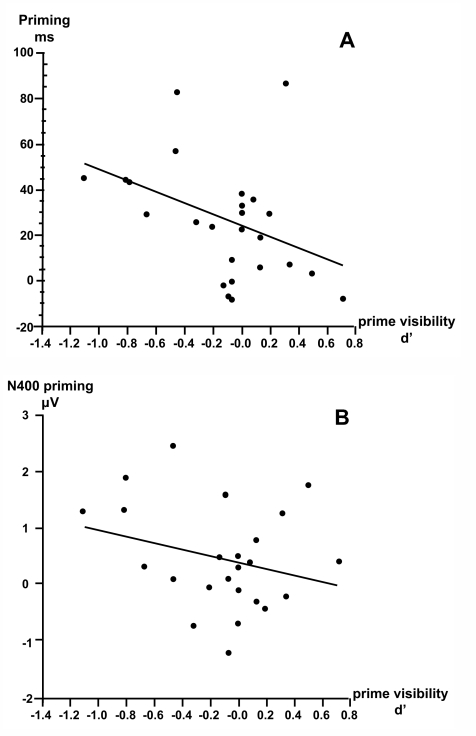
Plots of (**A**) masked behavioral and (**B**) masked parietal N400 priming effects as a function of the sensitivity measure d’ in the masked visibility test. The plots also show the linear regression function (after Kiefer, 2002b).

In a further study, Kiefer (2002b) took several measures to ensure that behavioural and N400 masked semantic priming effects indeed reflect unconscious automatic processes and are not compromised by conscious prime identification. In the first experiment, masked priming effects were related to recognition accuracy in a masked prime identification test (lexical decision on masked words and pseudowords) using a regression approach similar to that of Greenwald, Draine, and Abrams (1996). Kiefer (2002b) did not find a positive relation between the magnitude of priming effects and masked prime identification, thus ruling out the possibility that masked priming effects were contaminated by conscious prime identification. In fact, as can be seen in Figure 2, the correlation was clearly negative for behavioural priming effects suggesting that priming effects were greater the less conscious information could be obtained from the masked words (for a similar effect, see Carr & Dagenbach, 1990).

A negative correlation between d’ and the behavioral effect does not necessarily indicate that less discrimination abilities translate into stronger priming effects because large negative d’ values could indicate inverse response mapping. It should be noted however that in this study d’ were distributed around zero and negative values were small. Nevertheless, the correlation with priming was negative. For that reason, the negative values most likely reflect a random distribution around zero rather than inverse mapping of discriminated features.

In the second experiment, it was assessed whether masked stimuli could be recognised at the visual, lexical and semantic level and whether backward priming from the target to the prime had rendered the masked words partially recognisable. For instance, participants could have correctly completed the partially recognized prime word “t_ _ le” (“table”) in the context of the semantically related target word “chair”. To this end, subjects were required to perform decisions on visual, lexical and semantic features of masked words presented with or without semantically related context words. Subjects performed at chance level in all tasks (see Table 1). Most importantly, performance did not differ depending on whether the context word was related to the prime or not. These results exclude the possibility that backward priming has rendered the masked words partially visible.

**Table 1.** Identification measures for the masked stimuli as a function of task and semantic context (standard deviations in parentheses). Table after Kiefer (2002b).

	lexical decision without context	lexical decision with context	visual discrimination with context	semantic judgment
Average accuracy in %	50.8 (4.4) range 43.8 - 63.8	49.4 (2.9) range 44.4 - 56.3	49.9 (2.4) range 44.4 - 53.8	51.9 (5.7) range 44.4 - 65.0
Average d'	0 (.42)range -1.34- .74	related: -.15 (.37) range -1.34 - .42	related: 0 (.25) range -.55 - .39	.14 (.36) range -.41 - .89
		unrelated: 0 (.24)range -.42 - .39	unrelated: 0 (.16)range -.32 - .39	

## Classical and refined theories of automaticity

So far, I have shown that semantic meaning can be extracted from unconsciously perceived masked words in an automatic fashion. In this section, I will review different theories on the nature of automatic processes. Unconscious ‘automatic’^1^ processes are typically thought to be elicited autonomously and independently of any cognitive resources and intentions (Posner & Snyder, 1975; Schneider & Shiffrin, 1977). In classical theories of attentional control and automaticity, automatic processes are considered to be independent of capacity-limited attention in contrast to controlled processes (Posner & Snyder, 1975): Controlled processes are proposed (i) to depend on capacity-limited attentional resources, (ii) to interfere with other processes, (iii) to be executable only serially, and (iv) to be conscious. In contrast, it is assumed that automatic processes (i) do not depend on capacity-limited attentional resources, (ii) are not prone to interference with other processes, (iii) can work in parallel, and (iv) are unconscious (for a review, see Neumann, 1984). Hence, unlike controlled processes automatic processes are considered to be entirely autonomous from the configuration of the information processing system.

Neumann (1984) questioned these classical defining criteria of automatic processes. Instead, he proposed that automatic processes depend on a person’s current intentions and direction of attention. Furthermore, Neumann (1984) argued that automatic processes are prone to interference from other processes to some extent. Neumann (1984) assumed that the cognitive system has to be configured in a certain way or, as he calls it, “a variety of process parameters have to be specified for automatic processes to occur”. In his theory of direct parameter specification (DPS), which aims at explaining unconscious response priming, Neumann (1990) argues that participants’ search for information in order to specify free parameters within the currently active intention/action plan. Unconsciously registered information that resembles this searched-for information is selected and processed to specify the free processing parameters. Hence, according to DPS theory, masked response priming effects should depend on participants’ current intentions and action plans (for corresponding evidence see below).

The role of attention for eliciting automatic priming processes is also emphasized by Naccache, Blandin, and Dehaene (2002). They propose that automatic priming depends on a temporal window of attention which is open for a few hundreds of milliseconds when subjects focus their attention on the predicted time point of the appearance of a stimulus. Temporal attention is assumed to amplify the processing of the masked primes even if they are not consciously perceived. This top-down attentional amplification of unconsciously perceived masked primes enhances, in turn, the elicited automatic processes (see also Dehaene & Naccache, 2001). Naccache, Blandin, and Dehaene (2002) conclude that the concept of ‘automaticity’ has to be refined since unconscious, automatic processes appear to be modulated by top-down strategic control (for empirical evidence, see the section below). However, unconscious processing of the prime is automatic inasmuch as it cannot serve as a source of information for determining strategic processing steps (Merikle, Joordens, & Stolz, 1995).

In line with Neumann (1984) and Naccache, Blandin, and Dehaene (2002), I assume that attention and intentions configure the cognitive/neural system in a specific way (Kiefer & Brendel, 2006). A given attentional (or intentional) state might be necessary for unconscious stimuli to trigger further processes. These processes are not under intentional control once initiated and in that sense automatic (for a taxonomy of unconscious automatic processes, see Dehaene, Changeux, Naccache, Sackur, & Sergent, 2006). The proposed role of top-down attentional influences on unconscious automatic processing can indirectly be derived from a model of visual masking (Di Lollo, Enns, & Rensink, 2000; Enns & Di Lollo, 2000), which is based on re-entrant processing of visual stimuli. Di Lollo and Enns propose that visual stimuli are processed in a recurrent fashion in visual brain areas (V1, V2, V4 etc.): Activity in early visual areas is propagated to higher level areas and fed back to early visual areas (re-entrant processing). A conscious percept of the stimulus is achieved when re-entrant processing of a stimulus results in a stable activation pattern after several processing cycles.

As the mask interferes with the processing of the stimulus, a stable activation pattern is never reached even after many processing cycles. Enns and Di Lollo (2000) suggest that in addition to the amount of interference caused by competing stimuli (i.e., masks) attention is a crucial factor for whether or not re-entrant processing leads to a stable activation pattern representing the stimulus. Attention is thought to amplify the activation of the stimulus representation irrespective of whether or not a stable representation is achieved after several processing cycles. I therefore propose that attention is able to enhance the processing of both consciously and unconsciously perceived stimuli. In support of this view, Kentridge, Heywood, and Weiskrantz (2004) observed in patients with blindsight that spatial cueing improved discrimination performance without awareness (see also Kentridge, Heywood, & Weiskrantz, 1999). Thus, attention and conscious experience are functionally independent to some extent and should not be equated as some authors do (Merikle & Joordens, 1997; Velmans, 1991). Attention is obviously a prerequisite for conscious perception (Enns & Di Lollo, 2000; for a discussion also see Kiefer, 2002a). However, as argued here, allocation of attention might also be necessary for unconscious stimuli to trigger automatic processes.

## Top-down modulation of automatic processes: A gating framework

In this section, I want to expand the notion of a top-down modulation of automatic processes. In particular, I propose that automatic processes, which can be elicited by both consciously and unconsciously perceived stimuli, and controlled processes only acting upon consciously perceived stimuli are modulated by similar top-down influences. However, top-down modulation of processes elicited by consciously and unconsciously perceived stimuli presumably differs with regard to its temporal onset. As suggested by Ansorge and Horstmann (2007) I distinguish between two types of top-down control: preemptive and reactive control. In preemptive control, top-down influences are set up in advance of stimulus presentation. Preemptive control can be exerted for both conscious and unconscious stimulus presentation. However, only consciously perceived stimuli are susceptible to reactive control in response to ongoing or completed stimulus processing. For that reason, conscious ‘strategic’ stimulus processing allows for a greater adaptability and flexibility of top-down control than unconscious ‘automatic’ processing although both forms of processes share basic principles of top-down modulation. Given that automatic processes depend on the configuration of the cognitive system, one may also speak of “conditional automaticity” (Bargh, 1989; Logan, 1989) because automatic processes are not entirely bottom-up and stimulus driven, but are susceptible to top-down modulation. 

As outlined in the previous section, refined theories of automaticity suggest that the cognitive system has to be configured in a certain way for automatic processes to occur. The DPS theory (Neumann, 1990) suggests that attention, intentions, and task goals specify the necessary “parameters” within the information processing system so that an unconscious stimulus suffices to specify the remaining “free” parameters and to trigger a prepared response. But how could the “specification of process parameters” be implemented in a more formal, neuronally plausible mechanism? How could the notion of “parameter specification” be re-formulated in a more general way so that this concept is applicable not only to visuo-motor response preparation, but also to other domains such as semantic processing?

In the research on attention, the modulatory influences of attention on sensory processes are frequently assumed to be realised by a gating mechanism which enhances some processes while blocking others (Hamker, 2005). Attentional control is thought to be exerted by dorsolateral prefrontal areas, which mediate the representation of task-relevant information (i.e., task-relevant stimulus dimensions, spatial location, and temporal information of a stimulus). Sensory processing can be modulated by attention through far reaching neural connections from prefrontal areas to posterior brain areas (occipital and temporal cortex), in which the different stimulus dimensions are perceptually processed. Processing of task-relevant stimulus information is facilitated whereas processing of task-irrelevant information is blocked. This can be modeled by increasing the “gain” of neurons in brain areas which process task-relevant stimulus information while decreasing the gain of neurons in other areas (e.g., Cohen & Servan-Schreiber, 1992; Hamker, 2005). The gain is a parameter in neural network modeling which influences the probability that a neuron fires at a given activation level. If the gain is high the likelihood of firing is increased in comparison to a low gain. Through regulating the gain of sensory neurons, prefrontal areas could enhance sensory processing of task-relevant stimulus information and block the processing of task-irrelevant information. Electrophysiological animal studies, in which single or multiple cell activity was recorded, found neural response properties which are in line with the notion of an attentional gain control mechanism (Treue & Martínez Trujillo, 1999). The concept of gating by gain modulation introduced so far does not include a mechanism which actively inhibits task-irrelevant information. Instead, processing of task-irrelevant information is merely blocked (i.e. not carried out) by decreasing the gain in the corresponding neurons. The notion of blocking of information processing is in line with the available evidence presented below in the next section. To date, evidence does not support an active top-down inhibition of task-irrelevant processing pathways. For the sake of parsimony and due to the lack of supporting evidence, gating is solely realized through gain control in the proposed framework. Future work is clearly necessary to further elucidate the fine-grained details of the gating mechanism.

Similar to the present proposal, Stolz and Besner (1996) modeled within a connectionist network the influence of task sets on (unmasked) semantic priming effects (for the influence of task sets on semantic priming, see also the next section). In their model, a semantic layer is reciprocally connected with a lexical layer. Semantic priming occurs when activity in the semantic layer is fed-back to the lexical layer. They assume that a perceptual task orientation towards the prime (e.g., a letter search) blocks spreading activation from the semantic to the lexical layer hereby reducing or eliminating semantic priming effects. 

**Figure 3.**
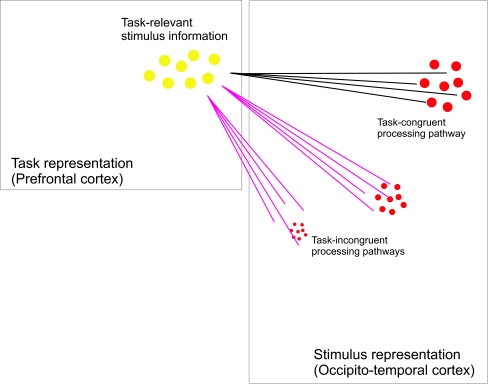
Outline of the gating framework. Task information (relevant stimulus dimensions, spatial and temporal stimulus information etc.) held in prefrontal areas modulates the gain of neurons in sensory areas through far reaching connections. Hereby, processing pathways in congruency with the represented task information are enhanced while other processing pathways are inhibited.

Gating mechanisms have been originally proposed for explaining effects of attention on the processing of visible stimuli which enter conscious awareness. However, the gating mechanism could also apply to unconscious perception and automatic processing. In particular, it can be used to model the modulatory effects of attention, intention and task sets on ‘automatic’ processes as suggested by refined theories of automaticity. I propose that the configuration of the cognitive system (or parameter setting) by attention, intention, and task sets is achieved by a similar kind of gating mechanism as suggested for conscious perception (see Figure 3). This gating mechanism orchestrates the information processing streams in congruency with the current task-representations even when perception is unconscious and processes are automatic. Unconsciously perceived masked stimuli can only trigger specific automatic processes (e.g., semantic priming) if the current task information held in prefrontal cortex gates the corresponding information processing pathway in posterior (semantic) brain areas. Otherwise, if the gating mechanism emphasizes other processing pathways, unconsciously perceived stimuli will not be able to elicit further ‘automatic’ processing. In line with the re-defined theories of automaticity described in the previous section, processes elicited by unconsciously perceived stimuli are automatic in the sense that they are not susceptible to top-down modulation or correction once the process has started (reactive control). Automatic processes can only be influenced by top-down modulation through gating before the process has started (preemptive control): The gating mechanism can configure the system in such a way that unconsciously perceived stimuli can elicit further processing steps in specific brain areas or it can block these processes. Of course, as the eliciting stimuli themselves are unconsciously perceived, top-down modulation cannot be exerted intentionally in deliberate anticipation of the stimuli (e.g., the masked prime in a masked priming paradigm). Instead, top-down modulation is induced indirectly by previous reactions, current intentions, stimuli, or task instructions and has to be set up in advance of stimulus presentation. According to my view, the possibility of (i) intended (ii) reactive (in response to ongoing or completed stimulus processing) top-down modulation remains to be the most prominent distinguishing feature between – what one might still call – controlled and automatic processes. In the next section, I describe studies which provide evidence for a top-down modulation of automatic processes elicited by unconsciously perceived stimuli.

## Evidence for top-down modulation of automatic processes

**Figure 4.**
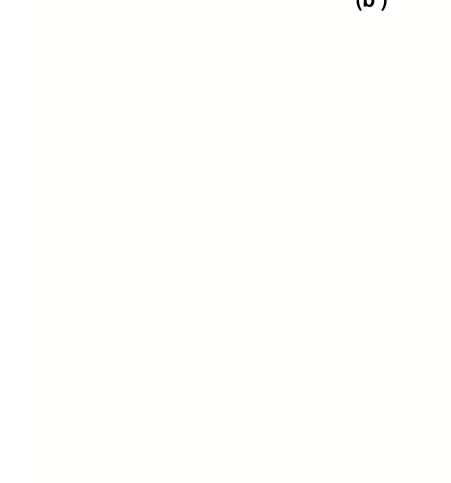
Schematic depiction of sample congruent and incongruent trials (a) and response times for the three conditions (b) in Experiment 1 of the Naccache et al. (2002) study. The motor response was congruent when the prime and the target numbers were both either greater than 5 or less than 5; if one was greater than 5 and the other was less than 5, they were incongruent. Response priming effects were only obtained when the target was presented after a fixed time interval (after Naccache et al., 2002).

In the first two studies reviewed in this section, the modulatory influence of temporal attention on automatic processes was investigated. These studies show that allocation of temporal attention is a prerequisite for automatic priming to occur. In all masked priming studies described in the first section of this article, subjects typically attended to the stimulation stream during the time windows of prime and target presentation. For that reason these earlier studies are not suitable to assess the influence of attention on automatic processes. Naccache, Blandin, and Dehaene (2002) manipulated in a numerical response priming paradigm the allocation of temporal attention to the target. In this paradigm (Dehaene et al., 1998), subjects were instructed to compare target numbers to a fixed reference of five. Numbers smaller and larger than five were assigned to different response hands. Subjects were faster when the masked prime and the target number fell on the same side of five, and therefore called for the same motor response than when they called for a different response (response priming effect, see also Vorberg, Mattler, Heinecke, Schmidt, & Schwarzbach, 2003). In order to manipulate the allocation of temporal attention, Naccache et al. (2002) presented subjects with a continuous stream of visual masks within which the primes and targets appeared at varying time points after trial onset. They compared the amount of priming on the same trials, depending on whether the time of target occurrence was blocked, and therefore predictable (implicit cueing), or variable, and therefore unpredictable (Experiment 1). They found response priming effects only when the onset of the target was predictable (Figure 4). In two more experiments temporal attention was explicitly cued, yielding identical results as with the implicit cuing procedure.

The Naccache et al. (2002) study provides supportive evidence for an attentional modulation of unconscious, automatic processes, but also has some limitations. First of all, attention was only cued to the appearance of the target. As primes and targets were presented in close temporal proximity, the prime also was attended to. However, attention to the prime and to the target is confounded. Therefore, the conclusion that temporal attention enhanced response priming effects by amplifying processing of the masked prime is not warranted, and the alternative interpretation that attentional enhancement of the target is a prerequisite for masked response priming cannot be ruled out. Secondly, Naccache et al. (2002) investigated the effects of temporal attention on response priming. It has been debated in the response priming literature whether masked response priming effects are mainly due to direct motor specifications without mediation through semantic processes (Abrams & Greenwald, 2000; Damian, 2001). For this reason, it is unclear whether the Naccache et al. (2002) results also hold for semantic priming. There is at least some evidence that unconscious behavioural semantic priming does not depend on spatial attention (Fuentes, Carmona, & Agis, 1994). However, this previous study only assessed behavioural priming, but did not record ERPs, so that it is open whether neurophysiological measures would be more sensitive to detect top-down attentional modulation of unconscious, automatic semantic priming.

**Figure 5.**
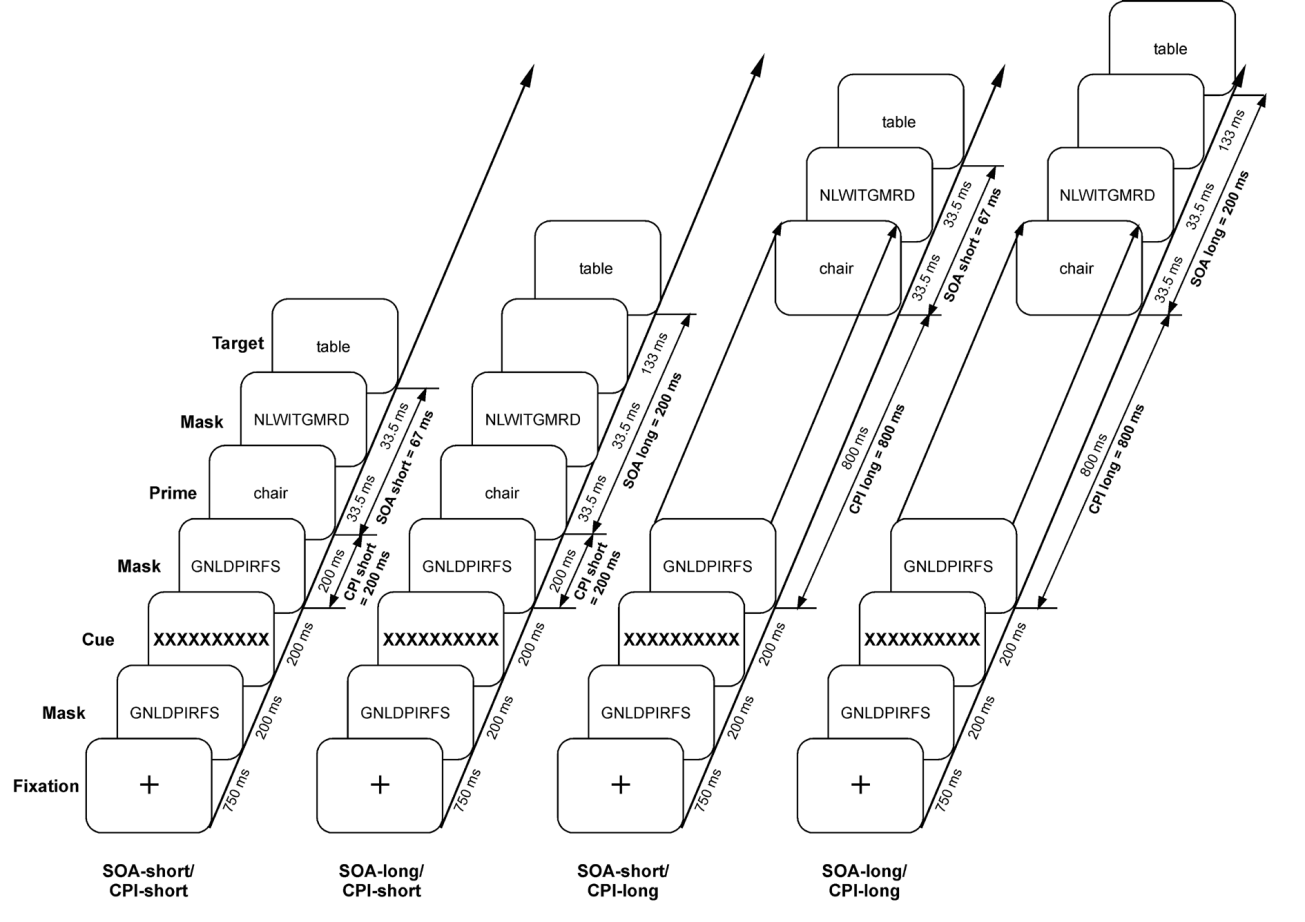
Temporal sequence of one trial of the temporal cueing procedure. The masked prime word was presented either 200 ms or 800 ms following a cue, which prompted subjects to attend to the stimulation stream (after Kiefer and Brendel, 2006).

Kiefer and Brendel (2006) set up a masked semantic priming paradigm, using ERPs to test whether temporal attention to the masked primes modulates behavioural and N400 priming effects. For the masked semantic priming paradigm, we adopted the design from our earlier studies (Kiefer, 2002b; Kiefer & Spitzer, 2000): Subjects performed lexical decisions on target stimuli (words and pseudowords), which were preceded by briefly presented (33.5 ms) masked prime words, which could not be consciously identified. In order to track the time course of masked priming, the prime-target SOA was either short (67 ms) or long (200 ms). In the first experiment, a cuing procedure was applied (see Figure 5) in order to prompt subjects to attend to the stimulation stream of masks either during the time window of masked prime presentation (short cue prime interval, CPI: 200 ms; plus 200 ms cue duration) or 1 s before masked prime presentation (long CPI: 800 ms; plus 200 ms cue duration). Filler trials with an intermediate CPI induced smoother transitions between trial lengths. In the long CPI condition, as a long period of time, during which the stimulation did not change, had elapsed after cue presentation, subjects should have disengaged temporal attention when the masked prime is finally presented. The combinations of CPI/SOA conditions were presented in a randomized sequence in order to prevent subjects from predicting the occurrence of the prime. Thus, in contrast to the Naccache et al. (2002) study, attention to the masked prime, not attention to the target, was manipulated. A second experiment was set up to control for whether possible interactions between masked priming and CPI did depend on attentional cuing to the prime or were merely the result of the different trial lengths. In this control experiment, the experimental procedure was the same except that participants were instructed to focus on the lexical decision on the target while the cue stimulus was not task-relevant. Analysis of reactions times showed that the manipulation of temporal attention to the prime in the first experiment was successful. In this experiment, in which the cue was task-relevant, slower reactions to the target in the short CPI condition demonstrated that participants focused attention to the stimulation stream immediately following cue presentation and had to re-allocate attention when the target was presented. In contrast, in the control experiment, in which the cue was task-irrelevant, we did not observe any RT differences as a function of the CPI.

**Figure 6.**
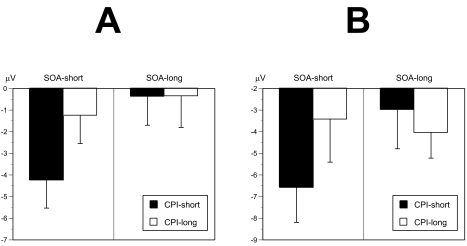
Attentional modulation of ERP priming effects. Mean voltages from centro-parietal electrodes in the time window (A) of the descending N400 (200-399) and (B) of the N400 peak (400-599 ms) as a function the cue prime interval (CPI) and prime-target SOA (Experiment 1). Voltages were collapsed across electrode sites. In both time windows N400 priming effects were largest at the short CPI/short SOA condition demonstrating an attentional modulation of masked semantic priming (after Kiefer and Brendel, 2006).

Kiefer and Brendel (2006) found that masked N400 priming effects had an earlier onset and were stronger in amplitude when primes were presented within the attended time window (short CPI) and when the prime-target SOA was short (67 ms) compared to the other conditions (Experiment 1). At the long SOA of 200 ms and when the prime was unattended (long CPI), the onset of the masked priming effect was delayed and N400 priming was generally smaller than in the short SOA/short CPI condition (see Figure 6). In Experiment 2, when subjects were instructed to focus upon the target, masked N400 priming was generally reduced such that it did not reach statistical significance at all. Taken together, this study provides strong evidence that attention to an unconsciously perceived masked stimulus is a prerequisite for N400 ERP priming effects to occur. The data therefore support the view that unconsciously perceived masked stimuli require attentional amplification to elicit automatic processes (Dehaene & Naccache, 2001; Naccache et al. 2002). It should be noted that in earlier masked priming studies (Deacon et al., 2000; Kiefer, 2002b; Kiefer & Spitzer, 2000), in which masked N400 priming effects were obtained, participants attended to the prime because the prime was presented shortly after the fixation cross and in close temporal proximity to the target.

Kiefer and Brendel (2006) were able to identify two important boundary conditions for obtaining reliable N400 priming effects: prime-target SOA and attention to the prime. Whatever the precise semantic process is that is indexed by N400 amplitude modulation, e.g., automatic spread of activation in semantic networks, it also occurs under automatic processing conditions (in addition to strategic processing conditions). However, automatic semantic processing decays fast over time when elicited by masked stimuli and requires temporal attention to the eliciting stimulus. 

The boundary conditions for masked N400 priming effects identified in this study may help to reconcile some discrepant findings in the literature regarding the processing nature of the N400. On the one hand, N400 amplitude has been shown to be modulated by unconsciously perceived masked words (Deacon et al., 2000; Kiefer, 2002b; Kiefer & Spitzer, 2000) and by words not available for verbal report during the attentional blink (Luck, Vogel, & Shapiro, 1996; Rolke, Heil, Streb, & Henninghausen, 2001; Vogel, Luck, & Shapiro, 1998), suggesting that the N400 ERP component is sensitive to automatic priming processes. On the other hand, N400 priming effects have only been found for attended, but not for ignored prime words (e.g., Bentin, Kutas, & Hillyard, 1995; Kellenbach & Michie, 1996). These latter findings have been taken as evidence that the N400 only reflects strategic post-lexical matching processes, but not automatic priming (e.g., automatic spreading of activation). The Kiefer and Brendel (2006) data allows to resolve this discrepancy. The observation of attentional modulation of unconscious masked N400 priming effects demonstrates that also automatic and not only strategic N400 priming requires that participants attend to the prime stimulus. 

Automatic priming does not only depend on temporal attention, but also on intentions and task sets, which are active during the presentation of the masked prime. In line with Rogers and Monsell (1995), I define task sets as an adaptive configuration of the cognitive system which is necessary to efficiently perform a given task (see also Gilbert & Shallice, 2002). The concept of a “task set” is related to that of “intention”, but is more specific because it refers to the immediate computational consequences of pursuing a current goal during task performance. The concept of “intention” is broader because it additionally includes the conscious representation of the goal and the subjective state of commitment to perform a goal-related action (Ansorge & Neumann, 2005; Goschke, 2002). 

For response priming, it is meanwhile well documented that response congruency effects (prime and target share the same or different responses) critically depend on participants’ intentions and expectations. Ansorge and colleagues (Ansorge, Heumann, & Scharlau, 2002; Ansorge & Neumann, 2005) showed in several studies that unconsciously perceived masked primes trigger responses only to the extent that they match currently active intentions of a person. When task instructions where changed in such a way that primes ceased to be task-relevant, priming effects were abolished. For instance, black-coloured primes elicited a response congruency effect on reactions to the target if the target was also shown in black colour. In contrast, when participants had to respond to red-coloured targets, black-coloured primes did not influence reaction times to the target anymore. In line with the DPS theory, Ansorge and Neumann (2005) argue that masked response priming effects depend on the formation of action plans: Participants search for information in order to specify free parameters within the currently active intention/action plan. Unconsciously registered information that resembles this searched-for information is selected and processed to specify the free processing parameters. Therefore, unconsciously perceived information will translate into behavioural effects that are absent if the same information is sufficiently dissimilar from the searched-for features. As the action plan has to be set up in advance of masked stimulus presentation, this situation is an instance of an exertion of preemptive control. Similar to Ansorge and colleagues, Eckstein and Perrig (2007), found masked response priming effects in semantic classification tasks only for word categories that matched participants’ current classification intention (e.g., living vs. non-living), but not for categories, which were irrelevant to their current classification intention (e.g., pleasant vs. unpleasant). 

In a related line of research, Kunde, Kiesel and Hoffmann (2003) investigated under which conditions novel masked primes, which do not belong to the target set, elicit response priming effects. As novel masked primes were never responded to during the course of the experiment, they cannot trigger a response based on simple S-R associations. Kunde et al. (2003) therefore assume that novel masked primes only elicit response priming effects when they are implicitly expected as release condition for a response (“action triggers”). In Experiment 1, novel masked primes were numerically embedded by the consciously presented target numbers (e.g., the primes 2 and 3 in the context of the targets 1 and 4) and thus implicitly expected as potential action triggers. In this experiment, reliable response priming effects were obtained for primes from the target set and also for novel primes. In Experiment 2, in contrast, novel prime numbers were not embedded by the target numbers (e.g., the primes 1 and 2 in the context of the targets 3 and 4) and were consequently not expected as action release conditions. In line with their assumptions, Kunde et al. (2003) observed response priming only for primes from the target set, but not for novel primes. The effects of intention and expectancy on masked response priming support the postulated gating framework. Intentions or expectations configure the cognitive system by establishing an intention-congruent processing pathway mapping a particular stimulus or stimulus dimension with a response and by blocking other pathways not matching the intention. As a consequence, only unconsciously perceived masked stimuli, which match current intentions, are able to trigger motor responses. 

While the influence of intentions on masked response priming is well documented, the effects of intentions or task sets on unconscious masked semantic priming have been rarely investigated. The dependency of semantic priming on intentions or task-sets is also less straight forward than for response priming, because semantic priming is based on highly overlearned associations between concepts and does not require the intention-based formation of S-R mappings during the course of the experiment. For that reason, the gating mechanism must serve a different purpose in semantic priming than in response priming although the basic principles may remain the same. In response priming, the gating mechanism is responsible for establishing a specific S-R mapping. In semantic priming, in contrast, the gating mechanism opens or blocks processing pathways dedicated to semantic stimulus processing. At present, evidence for a modulation of semantic priming by task sets comes mainly for visible prime processing: It has been shown that task sets imposed on prime processing modulate semantic priming effects even in conditions that emphasize automatic over strategic priming processes: When an orienting task does not require reading or semantic analysis of the prime, but instead a perceptual analysis of the letters forming the prime word, semantic priming is reduced or absent (Chiappe, Smith, & Besner, 1996; Mari-Beffa, Valdes, Cullen, Catena, & Houghton, 2005). Some studies even found semantic negative priming (e.g., Mari-Beffa, Houghton, Estevez, & Fuentes, 2000). These results are in line with the assumption of the gating framework proposed here: Task sets evoke a gating mechanism that enhances and blocks processing pathways, thereby optimizing task-related information processing.

It remains an open question whether such effects of task sets generalize to priming from unconsciously perceived masked words. With masked priming, the modulation of automatic semantic processing can be studied without any contamination by strategic mechanisms. In order to address this question, Kiefer (2006) modified the attentional cuing paradigm described above and presented a visible word either shortly before the masked prime (short CPI) or a longer time before (long CPI). Participants were instructed to perform two different tasks on these visible words in order to induce a semantic or perceptual task set prior to the presentation of the masked prime. Participants had to perform a semantic task on this word (living/non-living decision) or a perceptual task (Does the first/last letter of this word has a closed or open shape). Task switching studies showed that activated task sets persist for a longer time interval (Allport, Styles, & Hsieh, 1994; Meiran, 2000) and can even mediate unconscious response priming effects in the presence of a dominant competing task set (Kiesel, Kunde, & Hoffmann, 2007a). For that reason, it was assumed that the task set, which is induced by the first word (semantic vs. perceptual task set), would be active for a short period of time and could influence the processing of the subsequently presented masked prime through the gating mechanism postulated in the previous section. I therefore hypothesized that the induced task set is able to modulate masked priming effects. Only a semantic task set, but not the perceptual task set should open the processing stream for semantic analysis of the unconsciously perceived masked prime. Therefore, I expected masked semantic priming only when a semantic task was performed immediately before masked prime presentation. This prediction was largely upheld: Masked semantic priming effects in the behavioural and ERP data were largest for a semantic task set and smallest for a perceptual task set at the short CPI. At the long CPI, masked semantic priming effects recovered somewhat for the perceptual task set, but were reduced for a semantic task set, possibly due to an inhibition mechanism. As the tasks inducing the semantic and perceptual task sets differed with regard to general task difficulty, the results have to be considered as preliminary. Nevertheless, they are suggestive of the existence of a top-down gating mechanism which orchestrates the unconscious automatic processing stream in congruency with higher-level action goals and intentions.

Top-down control of automatic priming effects is also exerted when unconscious stimuli prime response tendencies that increase the probability of committing an error (Jaśkowski et al. 2003; Wolbers, Schoell, Verleger, Kraft, McNamara, Jaśkowski et al., 2006). In such a situation, top-down control is reactively engaged in response to the consciously perceived errors. However, with regard to the unconsciously perceived masked prime top-down control can be considered as preemptive because top-down mechanisms have to be set up in advance to masked prime presentation. Jaśkowski et al. (2003) found that the magnitude of masked response priming effects depended on the proportion of incompatible trials (i.e., trials in which prime and target were associated with different motor responses). A high proportion of incompatible trials, which increases the probability of committing an error, resulted in reduced masked response priming effects in comparison to a low proportion of incompatible trials. Jaśkowski et al. (2003) argue that unconscious response priming processes are under the observer’s strategic control, presumably as a function of the openly observable error frequency. ERP effects suggested that top-down control modulated sensory processing of the masked prime in the ventral pathway as well as response-related processing in the dorsal pathway. In line with the postulated gating framework outlined above, these findings suggest that a top-down gating mechanism is evoked when unconscious priming fosters unwanted response tendencies. This mechanism suppresses sensory prime processing as well as further automatic response preparation. 

## Future steps

The studies reviewed so far clearly show that automatic processes elicited by unconsciously perceived stimuli depend on a top-down configuration of the cognitive system. These findings support the assumption of refined theories of automaticity and are in clear contradiction with classical theories of automaticity which conceptualized automatic processes as being independent of cognitive resources and other top-down factors. The studies described in this article demonstrate that automatic processes depend on temporal attention, task sets and intention. I propose that these top-down influences on automatic processing can be accounted for by a gating framework which has been successfully applied to explain top-down attentional effects on the strategic processing of visible stimuli. Despite the considerable progress during the last years, we are only at the beginning of this new and exiting field of research. Future research is clearly needed to elucidate empirical phenomena and to develop a concise theory. I believe that the following steps have to be taken in future work.

At a theoretical level, the postulated gating framework needs further elaboration. The proposed gating mechanism which configures the cognitive system in congruency with the current goals and intentions has to be refined. In particular, formal computational modeling is required in order to ensure that the gating framework is indeed able to account for all empirical phenomena of top-down modulation. The neural network model by Hamker (2005) which has been developed to explain attentional modulation of sensory processing of visible stimuli might be a good starting point. In this context, the interesting question emerges whether a unitary type of gating mechanism is able to account for top-down modulation effects on visible stimuli as well as on unconsciously perceived masked stimuli. If so, this would suggest that conscious and unconscious perception is governed by the same set of processing principles.

At an empirical level, the generality of top-down modulation has to be determined. Firstly, it is an open question whether all different kinds of higher level factors discussed in the literature of attention and controlled processes (temporal and spatial attention, attention to stimulus dimensions, expectations, intentions, goals, task sets) exhibit similar modulatory influences on automatic processes. An answer to this question would not only elucidate top-down influences on automatic processing, it would also help to refine and to differentiate these partly interrelated concepts of top-down influences. Hence, although interesting in itself, the investigation of top-down modulations on automatic processes might also be used as a research tool to assess fine-grained consequences of top-down factors on the configuration of the information processing system.

Secondly, to date the influences of task sets on automatic processes elicited by unconsciously perceived stimuli have only been substantiated in masked semantic priming. However, it is not clear whether other forms of priming (response priming, attentional priming, perceptual priming, phonological priming) or automatic processes are similarly susceptible to modulation by task sets. At present, one study, which investigated the influence of task sets on masked response priming, failed to obtain any effect, admittedly under relatively specific dual task conditions (Experiment 3 of Ansorge, 2004). However, evidence for priming of task-sets could not be obtained. Conversely, the influence of intentions on automatic processes elicited by unconsciously perceived stimuli has only been assessed within the response priming paradigm so far (e.g., Ansorge et al., 2002; Ansorge & Neumann, 2005). It is possible that different forms of unconscious priming depend on automatic processing pathways which differ with regard to their sensitivity to top-down influences. This line of research would help to address the question whether or not automatic processes demonstrate the same properties irrespective of the involved cognitive and brain systems.

Thirdly, automatic processes can in principle be triggered by both unconsciously perceived and consciously perceived stimuli. It is an open question whether properties of automatic processes differ when triggered by consciously and unconsciously perceived stimuli, respectively. On the one hand there is evidence that automatic processes are governed by the same computational principles independent of whether they are triggered by unconsciously perceived or consciously perceived stimuli: At short SOAs, the time course of response priming is indistinguishable for consciously and unconsciously perceived primes suggesting similar underlying mechanisms (Vorberg, Mattler, Heinecke, Schmidt, & Schwarzbach, 2004). Moreover, automatic processes triggered by consciously perceived stimuli also seem to be modulated top-down: Interference effects which depend on the suppression of automated response tendencies such as the Stroop (Allport et al., 1994) or Simon effects (Hommel, 1993) vary as a function of participants’ intentions. On the other hand, top-down mechanisms might differ for conscious and unconscious stimulus presentations. As described above, conscious stimulus presentation allows for both preemptive and reactive control of stimulus processing whereas during unconscious stimulus presentation only preemptive control can be exerted. It should be noted, however, that it might be difficult to assess automatic processes in isolation by using consciously perceived stimuli. With consciously perceived stimuli a co-occurrence of both, automatic and strategic processes is probably the rule (Koivisto, 1998) rather than the exception (for a similar argument, see Jacoby, 1991).

Fourthly, the functional and neuroanatomical architecture of the postulated gating mechanism has to be further characterized. At a functional level, the more fine-grained details of the gating mechanism have to be specified. For instance, future research should clarify the possible contribution of active inhibition of task-irrelevant information to the gating mechanism. At a neurophysiological level, ERP studies are useful in order to determine the temporal course of top-down influences. Studies with functional magnetic resonance imaging (fMRI) are needed to identify the brain areas exerting top-down control (presumably prefrontal areas) and those being the target of control (presumably posterior sensory areas). While functional neuroimaging studies provide information at the system level, single cell recording studies in behaving animals can shed light on the fine-grained aspects of the postulated gating mechanism. In particular, they can provide information about response properties of neurons in sensory brain areas under different top-down influences. While gain modulation of visual neurons by attention has been documented (e.g. Treue & Martínez Trujillo, 1999), corresponding evidence with regard to the modulatory influences of goals, intention and task set is lacking so far. This information, in turn, may help to validate and to refine the proposed gating framework of top-down modulation.

## Footnotes

1 At this place, I enclose the word “automatic” in apostrophes in order to indicate that I do not use this term in the sense of classical theories of “automaticity”. For simplicity reasons, I omit the apostrophes in the remainder of the text.

## References

[R1] Abrams R. L., Greenwald A. G. (2000). Parts outweigh the whole (word) in unconscious analysis of
						meaning.. Psychological Science.

[R2] Allport A., Styles E. A., Hsieh S., Umilta C., Moscovitch M. (1994). Shifting intentional set: Exploring the dynamic control of
						tasks.. Attention and Performance 15: Conscious and Nonconscious Information
						Processing. Attention and Performance Series.

[R3] Anderson J. R., Bower G. H. (1973). Human associative memory..

[R4] Ansorge U. (2004). Top-down contingencies of nonconscious priming revealed by
						dual-task interference.. Quarterly Journal of Experimental Psychology.

[R5] Ansorge U., Heumann M., Scharlau I. (2002). Influences of visibility, intentions, and probability in a
						peripheral cuing task.. Consciousness and Cognition.

[R6] Ansorge U., Horstmann G. (2007). Preemptive control of attentional capture by colour: Evidence
						from trial-by-trial analyses and orderings of onsets of capture effects in
						reaction time distributions.. Quarterly Journal of Experimental Psychology.

[R7] Ansorge U., Neumann O. (2005). Intentions determine the effect of invisible metacontrast-masked
						primes: Evidence for top-down contingencies in a peripheral cueing
						task.. Journal of Experimental Psychology: Human Perception and
						Performance.

[R8] Ansorge U., Neumann O., Becker S., Kälberer H., Cruse H. (2007). Sensorimotor supremacy: Investigating conscious and unconscious
						vision by masked priming.. Advances in Cognitive Psychology.

[R9] Bargh J. A., Uleman J. S., Bargh J. A. (1989). Conditional automaticity: Varieties of automatic influence in
						social perception and cognition.. Unintended Thought.

[R10] Bentin S., Kutas M., Hillyard S. A. (1995). Semantic processing and memory for attended and unattended words
						in dichotic listening: Behavioral and electrophysiological
						evidence.. Journal of Experimental Psychology: Human Perception and
						Performance.

[R11] Bentin S., McCarthy G., Wood C. C. (1985). Event-related potentials, lexical decision, and se mantic
						priming.. Electroencephalography and clinical Neurophysiology.

[R12] Brown C. M., Hagoort P. (1993). The processing nature of the N400: Evidence from masked
						priming.. Journal of Cognitive Neuroscience.

[R13] Carr T. H., Dagenbach D. (1990). Semantic priming and repetition priming from masked words:
						Evidence for a center-surround attentional mechanism in perceptual
						recognition.. Journal of Experimental Psychology: Learning, Memory and
						Cognition.

[R14] Chiappe P. R., Smith M. C., Besner D. (1996). Semantic priming in visual word recognition: Activation blocking
						and domains of processing.. Psychonomic Bulletin & Review.

[R15] Chwilla D. J., Brown C. M., Hagoort P. (1995). The N400 as a function of the level of
						processing.. Psychophysiology.

[R16] Cohen J. D., Servan-Schreiber D. (1992). Context, cortex, and dopamine: A connectionist approach to
						behavior and biology in schizophrenia.. Psychological Review.

[R17] Damian M. F. (2001). Congruity effects evoked by subliminally presented primes:
						Automaticity rather than semantic processing.. Journal of Experimental Psychology: Human Perception &
						Performance.

[R18] Deacon D., Hewitt S., Chien-Ming Y., Nagata M. (2000). Event-related potential indices of semantic priming using masked
						and unmasked words: Evidence that the N400 does not reflect a post-lexical
						process.. Cognitive Brain Research.

[R19] Deacon D., Shelley-Tremblay J. (2000). How automatically is meaning accessed: A review of the effects of
						attention on semantic processing.. Frontiers in Bioscience.

[R20] Dehaene S., Changeux J. P., Naccache L., Sackur J., Sergent C. (2006). Conscious, preconscious, and subliminal processing: A testable
						taxonomy.. Trends in Cognitive Sciences.

[R21] Dehaene S., Naccache L. (2001). Towards a cognitive neuroscience of consciousness: Basic evidence
						and a workspace framework.. Cognition.

[R22] Dehaene S., Naccache L., LeClec’H G., Koechlin E., Mueller M., Dehaene-Lambertz G. (1998). Imaging unconscious priming.. Nature.

[R23] Di Lollo V., Enns J. T., Rensink R. A. (2000). Competition for consciousness among visual events: The
						psychophysics of reentrant visual processes.. Journal of Experimental Psychology: General.

[R24] Eckstein D., Perrig W. J. (2007). The influence of intention on masked priming: A study with
						semantic classification of words.. Cognition.

[R25] Enns J. T., Di Lollo V. (2000). What’s new in visual masking.. Trends in Cognitive Sciences.

[R26] Friederici A. D., Hahne A., Mecklinger A. (1996). Temporal structure of syntactic parsing: Early and late
						event-related potentials.. Journal of Experimental Psychology: Learning, Memory and
						Cognition.

[R27] Fuentes L. J., Carmona E., Agis I. F. (1994). The role of the anterior attention system in semantic processing
						of both foveal and parafoveal words.. Journal of Cognitive Neuroscience.

[R28] Gilbert S. J., Shallice T. (2002). Task switching: A PDP model.. Cognitive Psychology.

[R29] Goschke T., Müsseler J., Prinz W. (2002). Volition und kognitive Kontrolle. Lehrbuch der Allgemeinen Psychologie.

[R30] Greenwald A. G., Draine S. C., Abrams R. L. (1996). Three cognitive markers of unconscious semantic
						activation.. Science.

[R31] Hamker F. H. (2005). The reentry hypothesis: The putative interaction of the frontal
						eye field, ventrolateral prefrontal cortex, and areas V4, IT for attention
						and eye movement.. Cerebral Cortex.

[R32] Holcomb P. J., Neville H. J. (1990). Auditory and visual semantic priming in lexical decision: A
						comparison using event-related potentials.. Language and Cognitive Process.

[R33] Hommel B. (1993). Inverting the Simon effect by intention: Determinants of
						direction and extent of effects of irrelevant spatial
						information.. Psychological Research.

[R34] Hommel B., Monsell S., Driver J. (2000). The prepared reflex: Automaticity and control in
						stimulus-response translation.. Attention and performance 18: Control of Cognitive Processes. Attention
						and performance series..

[R35] Jacoby L. L. (1991). A process dissociation framework: Separating automatic from
						intentional uses of memory.. Journal of Memory & Language.

[R36] Jaśkowski P., Skalska B., Verleger R. (2003). How the self controls its “automatic pilot”
						when processing subliminal information.. Journal of Cognitive Neuroscience.

[R37] Kellenbach M. L., Michie P. T. (1996). Modulation of event-related potentials by semantic priming:
						Effects of color-cued selective attention.. Journal of Cognitive Neuroscience.

[R38] Kentridge R. W., Heywood C. A., Weiskrantz L. (1999). Effects of temporal cueing on residual visual discrimination in
						blindsight.. Neuropsychologia.

[R39] Kentridge R. W., Heywood C. A., Weiskrantz L. (2004). Spatial attention speeds discrimination without awareness in
						blindsight.. Neuropsychologia.

[R40] Kiefer M. (2001). Perceptual and semantic sources of category-specific effects in
						object categorization: Event-related potentials during picture and word
						categorization.. Memory & Cognition.

[R41] Kiefer M., Müsseler J., Prinz W. (2002a). Bewusstsein. Lehrbuch der Allgemeinen Psychologie.

[R42] Kiefer M. (2002b). The N400 is modulated by unconsciously perceived masked words:
						Further evidence for an automatic spreading activation account of N400
						priming effects.. Cognitive Brain Research.

[R43] Kiefer M. (2005). Repetition priming modulates category-related effects on
						event-related potentials: Further evidence for multiple cortical semantic
						systems.. Journal of Cognitive Neuroscience.

[R43a] Kiefer M. (2006). Top-down Modulation automatischer Prozesse durch
						Aufgabeneinstellungen..

[R44] Kiefer M., Brendel D. (2006). Attentional modulation of unconscious
						‘automatic’ processes: Evidence from event-related
						potentials in a masked priming paradigm.. Journal of Cognitive Neuroscience.

[R45] Kiefer M., Spitzer M. (2000). Time course of conscious and unconscious semantic brain
						activation.. NeuroReport.

[R46] Kiefer M., Weisbrod M., Kern I., Maier S., Spitzer M. (1998). Right hemisphere activation during indirect semantic priming:
						Evidence from event-related potentials.. Brain and Language.

[R47] Kiesel A., Kunde W., Hoffmann J. (2007a). Unconscious priming according to multiple S-R
						rules.. Cognition.

[R48] Kiesel A., Kunde W., Hoffmann J. (2007b). Mechanisms of subliminal priming.. Advances in Cognitive Psychology.

[R49] Klinger M. R., Burton P. C., Pitts G. S. (2000). Mechanisms of unconscious priming: I Response competition, not
						spreading activation.. Journal of Experimental Psychology: Learning, Memory and
						Cognition.

[R50] Klotz W., Neumann O. (1999). Motor activation without conscious discrimination in metacontrast
						masking.. Journal of Experimental Psychology: Human Perception and
						Performance.

[R51] Koivisto M. (1998). Categorical priming in the cerebral hemispheres: Automatic in the
						left hemisphere, postlexical in the right hemisphere?. Neuropsychologia.

[R52] Kunde W., Kiesel A., Hoffmann J. (2003). Conscious control over the content of unconscious
						cognition.. Cognition.

[R53] Kutas M., Hillyard S. A. (1980). Reading senseless sentences: Brain potentials reflect semantic
						incongruity.. Science.

[R54] Kutas M., Hillyard S. A. (1984). Brain potentials during reading reflect word expectancy and
						semantic association.. Nature.

[R55] Logan G. D., Uleman J. S., Bargh J. A. (1989). Automaticity and cognitive control.. Unintended thought.

[R56] Luck S. J., Vogel E. K., Shapiro K. L. (1996). Word meanings can be accessed but not reported during the
						attentional blink.. Nature.

[R57] Marcel A. J. (1983). Conscious and unconscious perception: Experiments on visual
						masking and word recognition.. Cognitive Psychology.

[R58] Mari-Beffa P., Houghton G., Estevez A. F., Fuentes L. J. (2000). Word-based grouping affects the prime-task effect on semantic
						priming.. Journal of Experimental Psychology: Human Perception &
						Performance.

[R59] Mari-Beffa P., Valdes B., Cullen D. J., Catena A., Houghton G. (2005). ERP analyses of task effects on semantic processing from
						words.. Cognitive Brain Research.

[R60] Meiran N., Monsell S., Driver J. (2000). Reconfiguration of stimulus task sets and response task sets
						during task switching.. Attention and performance 18: Control of Cognitive Processes. Attention
						and performance series.

[R61] Merikle P. M., Joordens S. (1997). Parallels between perception without attention and perception
						without awareness.. Consciousness and Cognition.

[R62] Merikle P. M., Joordens S., Stolz J. A. (1995). Measuring the relative magnitude of unconscious
						influences.. Consciousness and Cognition.

[R63] Milner A. D., Goodale M. A. (1995). The visual brain in action..

[R64] Naccache L., Blandin E., Dehaene S. (2002). Unconscious masked priming depends on temporal
						attention.. Psychological Science.

[R65] Neely J. H., Besner D., Humphreys G. W. (1991). Semantic priming effects in visual word recognition: A selective
						review of current findings and theories.. Basic progresses in reading – Visual word
						recognition..

[R66] Neumann O., Prinz W., Sanders A. F. (1984). Automatic processing: A review of recent findings and a plea for
						an old theory.. Cognition and motor processes.

[R67] Neumann O. (1990). Direct parameter specification and the concept of
						perception.. Psychological Research.

[R68] Nobre A. C., McCarthy G. (1995). Language-related field potentials in the anterior-medial temporal
						lobe: II. Effects of word type and semantic priming.. Journal of Neuroscience.

[R69] Posner M. I., Snyder C. R. R., Solso R. L. (1975). Attention and cognitive control.. Information processing and cognition..

[R70] Rogers R. D., Monsell S. (1995). Costs of a predictable switch between simple cognitive
						tasks.. Journal of Experimental Psychology: General.

[R71] Rolke B., Heil M., Streb J., Henninghausen E. (2001). Missed prime words within the attentional blink evoke an N400
						semantic priming effect.. Psychophysiology.

[R72] Scharlau I. (2007). Temporal processes in prime–mask interaction:
						Assessing perceptual consequences of masked information.. Advances in Cognitive Psychology.

[R73] Schneider W., Shiffrin R. M. (1977). Controlled and automatic human information processing: 1.
						Detection, search, and attention.. Psychological Review.

[R74] Stolz J. A., Besner D. (1996). Role of set in visual word recognition: Activation and activation
						blocking as nonautomatic processes.. Journal of Experimental Psychology: Human Perception &
						Performance.

[R75] Treue S., Martínez Trujillo J. C. (1999). Feature-based attention influences motion processing gain in
						macaque visual cortex.. Nature.

[R76] Vandenberghe R., Price C., Wise R., Josephs O., Frackowiak R. S. J. (1996). Functional anatomy of a common semantic system for words and
						pictures.. Nature.

[R77] Velmans M. (1991). Is human information processing conscious?. Behavioral and Brain Sciences.

[R78] Vogel E. K., Luck S. J., Shapiro K. L. (1998). Electrophysiological evidence for a postperceptual locus of
						suppression during the attentional blink.. Journal of Experimental Psychology: Human Perception and
						Performance.

[R79] Vorberg D., Mattler U., Heinecke A., Schmidt T., Schwarzbach J. (2003). Different time courses for visual perception and action
						priming.. Proceedings of the National Academy of Sciences, USA.

[R80] Vorberg D., Mattler U., Heinecke A., Schmidt T., Schwarzbach J., Kaernbach C., Schröger E., Müller H. (2004). Invariant time-course of priming with and without
						awareness.. Psychophysics beyond sensation: Laws and invariants of human
						cognition..

[R81] Wolbers T., Schoell E. D., Verleger R., Kraft S., McNamara A., Jaśkowski P. (2006). Changes in connectivity profiles as a mechanism for strategic
						control over interfering subliminal information.. Cerebral Cortex.

